# Influence of the Number of Axial Bexarotene Ligands on the Cytotoxicity of Pt(IV) Analogs of Oxaliplatin

**DOI:** 10.1155/2017/4736321

**Published:** 2017-07-19

**Authors:** Yulia N. Nosova, Ilia V. Zenin, Varvara P. Maximova, Ekaterina M. Zhidkova, Kirill I. Kirsanov, Ekaterina A. Lesovaya, Anna A. Lobas, Mikhail V. Gorshkov, Olga N. Kovaleva, Elena R. Milaeva, Markus Galanski, Bernhard K. Keppler, Alexey A. Nazarov

**Affiliations:** ^1^Department of Medicinal Chemistry and Fine Organic Synthesis, Lomonosov Moscow State University, Leninskie Gory 1/3, Moscow 119991, Russia; ^2^Blokhin Cancer Research Center, 24 Kashirskoye Shosse, Moscow 115478, Russia; ^3^Institute for Energy Problems of Chemical Physics, Russian Academy of Sciences, Leninsky Pr. 38, Bld.2, Moscow 119334, Russia; ^4^I.M. Sechenov First Moscow State Medical University, Moscow, Russia; ^5^Faculty of Chemistry, Institute of Inorganic Chemistry, University of Vienna, Waehringer Str. 42, 1019 Vienna, Austria

## Abstract

We present the synthesis and cytotoxic potencies of new Pt(IV) complexes with bexarotene, an anticancer drug that induces cell differentiation and apoptosis via selective activation of retinoid X receptors. In these complexes bexarotene is positioned as an axial ligand. The complex of one bexarotene ligand attached to Pt(IV) oxaliplatin moiety was potent whereas its counterpart carrying two bexarotene ligands was inactive.

## 1. Introduction

The discovery of anticancer properties of platinum based complexes became a significant breakthrough in cancer treatment [1]. Currently cisplatin, carboplatin, and oxaliplatin remain major drugs for the first line treatment (alone and in combination) for a variety of malignancies including head and neck, testicular, breast, and ovarian tumors [2–5]. However, despite the success of platinum containing drugs, the intrinsic or acquired resistance, general toxicity, and other severe side effects are clinically unfavorable [4–6]. To overcome these problems novel strategies for the search of active antitumor compounds are being developed. Octahedral Pt(IV) complexes are of interest because of their kinetic inertness, low general toxicity, and possibility for oral administration [7, 8]. Satraplatin was the first Pt(IV) compound to enter phase III clinical trials as an oral drug for treatment of hormone-refractory prostate cancer. Unfortunately, this compound showed no convincing benefit for overall patient survival and was not approved by the FDA. Still, clinical trials of satraplatin in combination with different organic drugs continued [9, 10].

Combinations of two drugs in one molecule are extensively used in modern drug discovery and allow for control of activity, selectivity, and pharmacokinetics. The synthetic advantage of Pt(IV) complexes is the suitability for chemical modifications of axial positions which makes introduction of new active compounds relatively easy. Based on the proposed mechanism of action for Pt(IV) complexes, that is, activation by reduction, the release of the axial ligands can be useful for drug targeting and delivery to cancer cells [11]. This approach yielded a variety of promising complexes containing axial ligands such as folic acid [12], estradiol [13], short peptides [14], inhibitors of glutathione-S-transferase [15], pyruvate dehydrogenase kinase [16–18], histone deacetylase [19–21], cyclooxygenase [22–24], mitochondria associated hexokinase [25], or p53 activators [26].

Retinoids are biologically active analogs of vitamin A, which play an essential role in cell proliferation, differentiation, and apoptosis. Bexarotene, a selective agonist of retinoid X receptors, is used to treat cutaneous T-cell lymphoma by inducing cell differentiation and apoptosis and inhibiting metastasis [27–29]. Recently we have shown that introduction of bexarotene into Ru(II)-arene compounds resulted in highly cytotoxic agents [30]. Here, we report the synthesis, chemical characterization, and antiproliferative activity of Pt(IV) complexes with covalently attached axial ligand bexarotene.

## 2. Experimental Section

### 2.1. Materials

(OC-6-33)-(*trans*-1R,2R-Diaminocyclohexane)dihydroxido(oxalato)platinum(IV) [31], (OC-6-44)-acetato(*trans*-1R,2R-diaminocyclohexane)hydroxido(oxalato)platinum(IV) [32], and bexarotene [33] were synthesized in the Laboratory of Bioorganometallic Chemistry, Moscow State University. Oxalyl chloride was purchased from Fluka, St. Louis, USA.

### 2.2. Physical Measurements

NMR spectra were recorded on a Bruker FT-NMR Avance III 500 MHz instrument at 500.32 (^1^H), 125.81 (^13^C), 50.70 (^15^N), and 107.57 (^195^Pt) MHz. 2D NMR measurements were carried out using standard pulse programs. Chemical shifts were referenced relative to the solvent signal for ^1^H and ^13^C spectra. For ^15^N and ^195^Pt spectra, the external standards NH_4_Cl and K_2_[PtCl_4_] were used. ESI mass spectra were recorded on a LC/MSn ion trap mass spectrometer amaZon SL (Bruker, Bremen, Germany) with MeOH as a solvent. Elemental analysis was performed at Moscow State University with MicroCube Elementar analyzer.

### 2.3. Cell Lines and Culture Conditions

The MCF7, MCF7D (gift of N. I. Moiseeva), HaCat, A549, and SW480 cell lines were cultured in Dulbecco modified Eagle's medium (DMEM; PanEco, Russia) with 10% fetal bovine serum (HyClone, USA) and antibiotics (PanEco, Russia) in 5% CO_2_, 37°C. The compounds were predissolved at 20 mM in dimethyl sulfoxide (DMSO) and added to the cell culture at the required concentration with maximum DMSO content of 0.5 v/v%. Cells in 96-well plates (7 × 10^3^ cells/well) were treated with various concentrations of** 3**,** 4**, cisplatin, or bexarotene at 37°C for 72 h. Cell viability was determined using the MTT assay as follows: cells were incubated at 37°C for 4 h with 20 *μ*l of 5 mg/ml solution of 3-(4,5-dimethylthiazol-2-yl)-2,5 diphenyltetrazolium bromide (Sigma-Aldrich, St. Louis, USA). The supernatant was discarded and formazan was dissolved in 150 *μ*l of DMSO. The optical density of the solution was measured at 550 nm on a multiwell plate reader (Multiskan FC, Thermo Fisher Scientific, USA). The percentage of viable (i.e., MTT converting) cells was calculated from the absorbance of untreated cells (100%). Each experiment was repeated three times, and each concentration was tested in three replicates.

### 2.4. Synthesis

#### 2.4.1. (OC-6-33)-(trans-1R,2R-Diaminocyclohexane)-bis(4-(1-(3,5,5,8,8-pentamethyl-5,6,7,8-tetrahydro-2-naphthyl)vinyl)benzoato)oxalatoplatinum(IV) 3

**Scheme 1:**
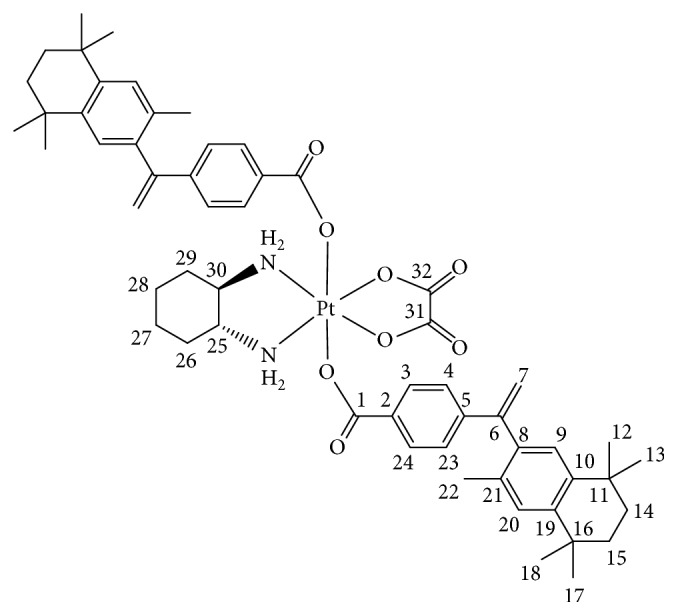


 In Scheme 1, oxalyl chloride (2.56 mL, 30.0 mmol) followed by one-two drops of DMF was added to a stirred suspension of 4-(1-(3,5,5,8,8-pentamethyl-5,6,7,8-tetrahydro-2-naphthyl)vinyl)benzoic acid (413 mg, 1.19 mmol) in CH_2_Cl_2_ (60 mL). The reaction mixture was refluxed for 1 h until a clear solution was formed and then solvent and unreacted oxalyl chloride were removed under reduced pressure to yield the acid chloride as a pale yellow solid that was used without purification. A solution of 4-(1-(3,5,5,8,8-pentamethyl-5,6,7,8-tetrahydro-2-naphthyl)vinyl)benzoyl chloride in acetone (40 mL) was added to a stirred suspension of (OC-6-33)-(trans-1R,2R-diaminocyclohexane)dihydroxido(oxalato)platinum(IV) (100 mg, 0.23 mmol) and pyridine (193 *μ*l, 2.4 mmol) in acetone (30 mL). The reaction mixture was stirred at room temperature for 12 h and concentrated to ~3 mL and the white precipitate formed was filtered off and washed with diethyl ether (2 × 5 mL). The compound was purified by column chromatography on Silicagel with acetone as eluent. The solvent was removed under reduced pressure; the compound was washed with diethyl ether (2 × 5 mL) and dried. Yield: 135 mg (53%), m.p. 213-214°C (decomp.). C_56_H_68_N_2_O_8_Pt (1091.46): calcd. C 61.58, H 6.28, N 2.56; found C 61.25, H 5.89, N 2.42. ^**1**^**Н NMR** ([d6]-DMSO) *δ*: 8.51 (d, 2Н, *J* = 6.2 Hz, NH_2_), 8.23 (t, 2Н, *J* = 9.1 Hz, NH_2_), 7.84 (d, 4Н, *J* = 8.5 Hz, H3, H24), 7.30 (d, 4Н, *J* = 8.5 Hz, H4, H23), 7.15 (s, 2Н, H20), 7.08 (s, 2Н, H9), 5.90 (s, 2Н, H7), 5.25 (s, 2Н, H7), 2.82–2.74 (m, 2Н, H25, H30), 2.17 (d, 2Н, *J* = 10.7 Hz, H26, H29), 1.89 (s, 6Н, H22), 1.66 (s, 8Н, H14, H15), 1.58–1.48 (m, 4Н, H26, H27, H28, H29), 1.27 (s, 12Н, H17, H18), 1.25–1.18 (m, 14Н, H12, H13, H27, H28) ppm. ^**13**^**C NMR **([d6]-DMSO) *δ*: 172.8 (C1), 164.5 (C31, C32), 148.8 (C6), 144.2 (C19), 144.2 (C5), 142.3 (C10), 138.4 (C8), 132.5 (C21), 132.1 (C2), 130.2 (C3, C24), 128.3 (C20), 127.8 (C9), 126.4 (C4, C23), 117.4 (C7), 61.6 (C25, C30), 35.1 (C14/15), 35.1 (C14/15), 34.1 (C11/16), 34.0 (C11/16), 32.1 (C12, C13/C17, C18), 32.1 (C12, C13/C17, C18), 31.2 (C26, C29), 24.0 (C27, C28), 20.0 (C22) ppm. ^**15**^**N NMR **([d6]-DMSO) *δ*: −3.9 (NH_2_) ppm. ^**195**^**Pt NMR **([d6]-DMSO) *δ*: 3228 ppm. ESI-MS:* m/z* = 1115 [M+Na^+^]^+^.

#### 2.4.2. (OC-6-44)-acetato(trans-1R,2R-Diamineocyclohexane)-4-(1-(3,5,5,8,8-pentamethyl-5,6,7,8-tetrahydro-2-naphthyl)vinyl)benzoato)oxalatoplatinum(IV) 4

**Scheme 2:**
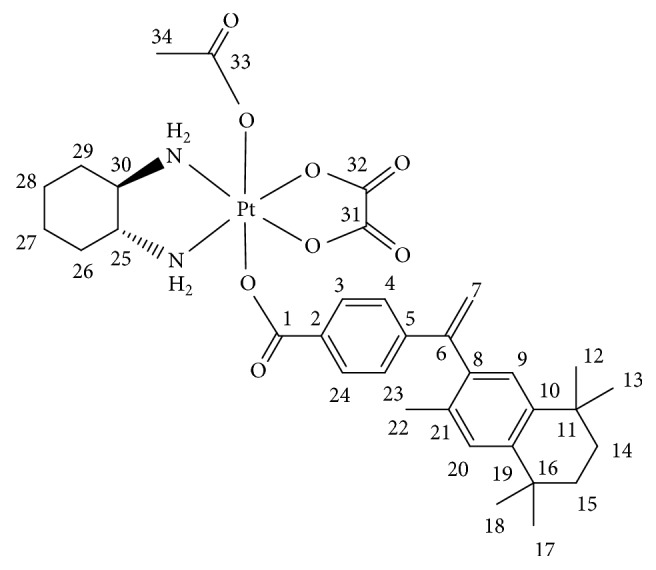


 In Scheme 2, oxalyl chloride (1.32 mL, 15.4 mmol) and a catalytic amount of DMF were added to a suspension of 4-(1-(3,5,5,8,8-pentamethyl-5,6,7,8-tetrahydro-2-naphthyl)vinyl)benzoic acid (214 mg, 0.62 mmol) in CH_2_Cl_2_ (40 mL). The reaction mixture was refluxed for 1 h until a clear solution was formed. Solvent and unreacted oxalyl chloride were removed under reduced pressure to yield the acid chloride as a pale yellow solid that was used without purification. A solution of 4-(1-(3,5,5,8,8-pentamethyl-5,6,7,8-tetrahydro-2-naphthyl)vinyl)benzoyl chloride in acetone (20 mL) was added to a suspension of (OC-6-44)-acetato(trans-1R,2R-diaminocyclohexane)hydroxido(oxalato)platinum(IV) (115 mg, 0.24 mmol) and pyridine (100 *μ*l, 1.24 mmol) in acetone (30 mL). The reaction mixture was stirred at room temperature for 12 h and a formed precipitate was filtered off, washed with diethyl ether (3 × 5 mL) and water (3 × 5 mL), and dried under reduced pressure. Yield: 70 mg (36%). m.p. 215-216°C (decomp.). C_34_H_44_N_2_O_8_Pt (803.81): calcd. C 50.80, H 5.52, N 3.49; found C 50.55, H 4.97, N 3.48. ^**1**^**Н NMR **([d6]-DMSO) *δ*: 8.44 (brs, 2Н, NH_2_), 8.38 (t, 1Н, *J* = 9.7 Hz, NH_2_), 8.13 (t, 1Н, *J* = 9.7 Hz, NH_2_), 7.82 (d, 2Н, *J* = 8.5 Hz, H3, H24), 7.28 (d, 2Н, *J* = 8.5 Hz, H4, H23), 7.14 (s, 1Н, H20), 7.07 (s, 1Н, H9), 5.89 (s, 1Н, H7), 5.24 (s, 1Н, H7), 2.77–2.65 (m, 1Н, H25/30), 2.66–2.57 (m, 1Н, H25/30), 2.16–2.10 (m, 2Н, H26, H29), 1.99 (s, 3Н, H34) 1.88 (s, 3Н, H22), 1.65 (s, 4Н, H14, H15), 1.55–1.41 (m, 4Н, H26, H27, H28, H29), 1.26 (s, 6Н, H17, H18), 1.23 (s, 6Н, H12, H13), 1.22–1.14 (m, 2H, H27, H28) ppm. ^**13**^**C NMR **([d6]-DMSO) *δ*: 179.0 (C33), 172.6 (C1), 164.2 (C31, C32), 148.8 (C6), 144.2 (C19), 144.1 (C5), 142.3 (C10), 138.4 (C8), 132.5 (C21), 132.2 (C2), 130.2 (C3, C24), 128.3 (C20), 127.8 (C9), 126.3 (C4, C23), 117.3 (C7), 61.8 (C25/30), 61.3 (C25/30), 35.1 (C14/15), 35.1 (C14/15), 34.1 (C16), 34.0 (C11), 32.1 (C12, C13/C17, C18), 32.1 (C12, C13/C17, C18), 31.3 (C26, C29), 24.0 (C27, 28), 23.5 (C34), 19.9 (C22) ppm. ^**15**^**N NMR **([d6]-DMSO) *δ*: −5.3 (NH_2_), −5.1 (NH_2_) ppm. ^**195**^**Pt NMR **([d6]-DMSO) *δ*: 3233 ppm. ESI-MS:* m/z* = 826 [M + Na^+^]^+^.

## 3. Results and Discussion

**Scheme 3:**
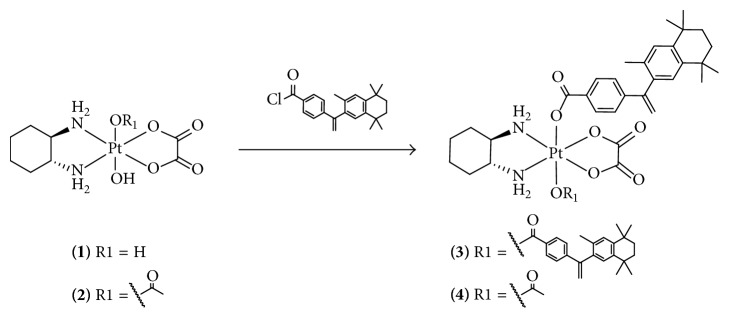
Synthesis of Pt(IV) complexes.

In order to obtain new Pt(IV) complexes with different number of bexarotene moieties as axial ligands, we used Pt(IV) analogs of oxaliplatin and the acid chloride of bexarotene (Scheme 3). Complexes** 3** and** 4 **were prepared by reacting an excess of 4-(1-(3,5,5,8,8-pentamethyl-5,6,7,8-tetrahydro-2-naphthyl)vinyl)benzoyl chloride (prepared in situ from bexarotene and oxalyl chloride) with (OC-6-33)-(*trans*-1R,2R-diaminocyclohexane)dihydroxido(oxalato) platinum(IV) or (OC-6-44)-acetato(trans-1R,2R-diaminocyclohexane)hydroxido(oxalato) platinum(IV), respectively. Pyridine was used as an acceptor of HCl. Crude complexes were precipitated after concentration of the reaction mixture. Pyridine hydrochloride was removed by washing the precipitate with water to yield the pure complex.

**Figure 1:**
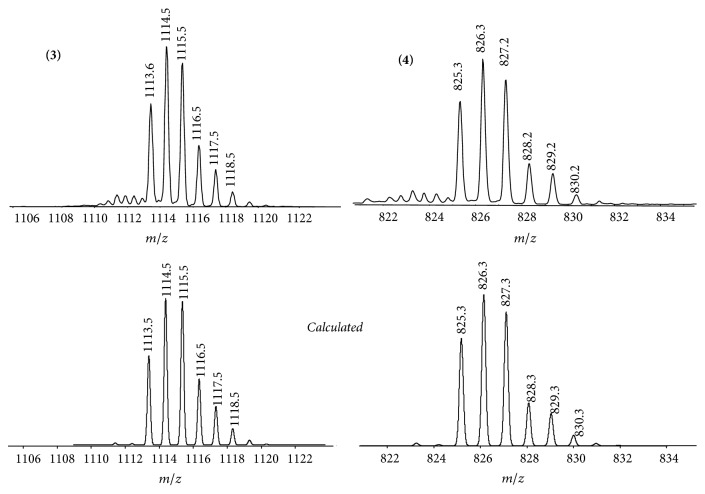
ESI-MS corresponding to [M+Na^+^]^+^ for complexes** 3** and** 4**.

Complexes** 3** and** 4** were characterized by ^1^H, ^13^C, ^15^N, ^195^Pt 1D, and 2D NMR spectroscopy, ESI mass spectrometry, and elemental analysis. In the ^13^C{^1^H} spectra we observed a shift of the carboxylic group that confirms the attachment of bexarotene to the platinum center. The nature of the axial ligand had only a minor influence on the ^1^H and ^13^C resonances in the oxaliplatin moiety [25, 36]. In ESI mass spectra the most abundant peaks were assigned to the [M+Na^+^]^+^ ion in the positive ion mode or to the [M−H^+^]^−^ ion in the negative ion mode for complex** 4**, respectively; additionally minor peaks can be assigned to proton and potassium adducts. For all mass spectra the experimental isotopic patterns were in a good agreement with calculated isotopic distribution (Figure 1).

**Figure 2:**
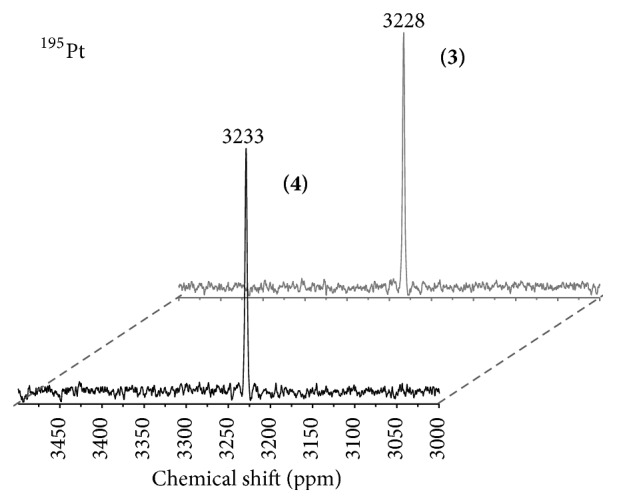
^195^Pt NMR complexes** 3** and** 4**.

^195^Pt NMR spectroscopy is a known method for monitoring the coordination sphere of the Pt(IV) center [34, 37]. For complexes** 3** and** 4**, the resonance at 3228 ppm and 3233 ppm, respectively, indicates a Pt(IV)N_2_O_4_ coordination sphere (Figure 2). As reported earlier, the nature of carboxylates in the axial position has no dramatic influence on the resonance in ^195^Pt spectra [25, 36, 37].

The cytotoxicity of complexes** 3** and** 4**, bexarotene, and cisplatin was evaluated in the human MCF7 breast cancer cell line and its doxorubicin/cisplatin resistant subline MCF7D, as well as against colon carcinoma SW480 cells, non-small cell lung carcinoma A549, and immortalized human nonmalignant keratinocyte HaCat cell line using the colorimetric MTT-test after 72 h of incubation (Table 1). The cytotoxic potency of oxaliplatin, the Pt(II) precursor of the new complexes, was taken from literature.

Complex** 3 **with two axial bexarotene ligands did not affect the viability of SW480, MCF7, MCF7D, and HaCat cells at concentrations < 100 *μ*M. Also, the cytotoxic effect against non-small cell lung carcinoma cell line A549 (IC_50_ = 83 ± 16 *μ*M) was minor. In contrast, complex** 4** with one bexarotene ligand showed a considerably higher cytotoxicity than cisplatin against SW480, HaCat, MCF7, MCF7D, and A549 cells. Complex** 4** was notably more active than parent bexarotene and exhibited high sensitivity against breast cancer cells: the IC_50_ value in the MCF7 cell line was in the submicromolar range, providing a promising basis for further investigation (Table 1). Such a specificity (complex** 4** is active, but** 3 **is not) is unexpected but rarely reported in the literature for Pt(IV) complexes with different ligands [37, 38], although it is not a general rule. Recently we presented a similar design with lonidamine as biologically active component and no such specificity was observed [25].

## 4. Conclusions

A novel platinum(IV) complex featuring the oxaliplatin core and one axial bexarotene ligand exhibited high cytotoxicity against a panel of tumor cell lines. This complex is more active than cisplatin and preferential sensitivity of a breast cancer cell line to** 4** compared to nonmalignant cells was found. Remarkably, complex** 3** with two bexarotene ligands showed no activity in the tested cell lines. These results reveal a good potential for the use of bexarotene as ligand in the search for new metal-based anticancer compounds.

## Figures and Tables

**Table 1:** Cytotoxicity of new complexes, bexarotene, cisplatin, and oxaliplatin.

Compounds	IC_50_ (*µ*M)^*∗*^				
	SW480	A549	MCF7	MCF7D	HaCat
**3**	>100	83 ± 16	>100	>100	>100
**4**	11 ± 1.6	10 ± 1	0.47 ± 0.07	4.8 ± 0.5	8 ± 1.3
Bexarotene	80 ± 10	85 ± 9	67 ± 13	71 ± 21	>90
Cisplatin	14 ± 4.4	29.0 ± 10	14 ± 7	75 ± 5.8	30 ± 10
Oxaliplatin	0.9 ± 0.3 [34]	11.5 ± 3.9 [34]	43.8 [35]	—	—

^*∗*^Data are mean ± standard deviation from 3 independent experiments; each drug concentration was tested in triplicate. —: no data.
